# Dating app use and unhealthy weight control behaviors among a sample of U.S. adults: a cross-sectional study

**DOI:** 10.1186/s40337-019-0244-4

**Published:** 2019-05-31

**Authors:** Alvin Tran, Christian Suharlim, Heather Mattie, Kirsten Davison, Madina Agénor, S. Bryn Austin

**Affiliations:** 1 Department of Nutrition, Harvard TH Chan School of Public Health, 665 Huntington Avenue, Boston, MA 02115 USA; 2 Department of Social and Behavioral Sciences, Harvard TH Chan School of Public Health, 677 Huntington Avenue, Boston, MA 02115 USA; 3 Center for Health and Decision Science, Department of Health Policy and Management, Harvard TH Chan School of Public Health, 718 Huntington Avenue, Boston, MA 02115 USA; 4 Department of Biostatistics, Harvard TH Chan School of Public Health, 655 Huntington Avenue, Boston, MA 02115 USA; 5 Division of Adolescent and Young Adult Medicine, Boston Children’s Hospital, 300 Longwood Avenue, Boston, MA 02115 USA

**Keywords:** Dating apps, Unhealthy weight control behaviors, Social media, Disordered eating, Online dating

## Abstract

**Background:**

Online dating has become increasingly popular over the years. Few research studies have examined the association between dating apps and disordered eating. In this study, we evaluated the association between dating app use and unhealthy weight control behaviors (UWCBs) among a sample of U.S. adults.

**Methods:**

Our sample includes 1769 adults who completed an online survey assessing dating app use and UWCBs in the past year. Survey assessed participants’ self-reported frequency of using dating apps within the past 30 days and engagement in six UWCBs with the purpose of lowering weight or changing their body shape within the past 12 months. UWCBs included vomiting, laxative use, fasting, diet pill use, muscle building supplement use, and use of anabolic steroids.

**Results:**

Results of multivariate logistic regression models suggest dating app users had substantially elevated odds of UWCBs compared with non-users (odds ratios [OR] range = 2.7—16.2). These findings were supported by results of additional gender-stratified multivariate logistic regression analyses among women and men.

**Conclusions:**

This study’s findings contribute to the limited literature exploring the association between dating app use and adverse health outcomes, particularly UWCBs. While additional longitudinal and representative research is needed, public health professionals ought to explore dating app use as a potential risk factor for UWCBs.

**Electronic supplementary material:**

The online version of this article (10.1186/s40337-019-0244-4) contains supplementary material, which is available to authorized users.

## Plain English summary

Dating app use is common among both men and women and these apps are often used to find romantic and sexual partners. They represent a growingly popular form of non-traditional media that provides a digital platform where people can evaluate others based on many attributes, including physical appearance. Despite their popularity, very little research has explored dating app use in relation to eating disorders and their risk factors. In this study, we assessed the cross-sectional association between dating app use and six unhealthy weight control behaviors (fasting, diet pill use, laxative use, self-induced vomiting, use of muscle-building supplements, and use of anabolic steroids) using an online survey completed by more than 1700 adults in the United States. Results showed that compared to non-users, those who used dating apps had significantly elevated odds of UWCBs.

## Background

Online dating has become increasingly popular in the United States (U.S.). Fifteen percent of U.S. adults say they have used online dating sites or mobile dating applications, or “dating apps”, in 2015 – a number up from 11% in 2013 [1]. Young adults, defined as those between ages 18- to 24-years old, as well as older adults, those in their 50s and 60s, contributed the most to this increase in dating app usage [1]. In addition, results from a 2017 survey suggest current dating app use could be as high as 30% among 18- to 29-year-old U.S. adults [2].

Mobile dating applications – commonly referred to as “dating apps” in popular culture – are designed to enable their users to locate potential romantic partners, friends, and other acquaintances [3]. And while they are primarily marketed as an avenue to find dates and potential romantic partners, motivations to use dating apps have evolved over time. For instance, people are using dating apps for socializing, to pass time, to improve their flirting and social skills, and to engage in casual sex [4–6]. Prior studies suggest that dating apps may serve as an avenue for members of sexual and gender minority groups (e.g., individuals who identity as gay, lesbian, bisexual, transgender) to meet without having to disclose their sexual orientation identity or attraction to others in a more public setting [7]. Regardless of sexual orientation identity, the majority of online dating users agree that dating digitally has many advantages over other ways of finding romantic partners, such as increased ease of use and efficiency, and likelihood of finding a better match [1].

Speculation has grown over the frequency of dating app use and its relationship with body image dissatisfaction. In a study of nearly 1000 participants, Strubel and Petrie (2017) compared body image concerns between users and nonusers of the dating app Tinder. They found that regardless of gender, Tinder users reported significantly lower levels of satisfaction with their faces and bodies and higher levels of internalization, appearance comparisons and body shame compared to non-users [8]. As with social media platforms, such as Facebook and Instagram, dating apps also allow people to connect, network and socialize with others, often providing an opportunity to see other users’ semi-public profiles and photos [4]. On Tinder [9], which has an estimated 50 million users worldwide and 10 million active daily users [10], users can “swipe right” or “swipe left” to indicate if they respectively like or dislike a particular profile [8]. Thus, individual dating app users are continuously engaging in a cycle in which they are evaluating profile pictures and brief descriptions of others yet are being subject to scrutiny themselves. Some research studies also suggest dating apps may provide new avenues for appearance-based discrimination among users [11]. Results from a content analysis of 300 profiles of a dating app primarily used by men who have sex with men suggest femmephobia, or anti-effeminate, language was common among users [11].

In general, the mass media has been linked to body image concerns [12]. Studies suggest that the mass media - from television, magazines, to social media – contributes to body dissatisfaction by perpetuating dominant body image ideals for men [13] and for women [14, 15]. For men, this culturally constructed, dominant ideal is often one that is generally muscular with little body fat [16]. For women, the thin-ideal is often the idealized social norm for the female body [17] although the pressure to achieve this ideal may vary across racial/ethnic groups [18, 19]. Such media-portrayed images, which often are mostly unattainable and unrealistic, may result in body dissatisfaction and lead to unhealthy weight control behaviors (UWCBs) [20], which include a constellation of dangerous behaviors, such as extreme food restriction (fasting), laxative use, self-induced vomiting, and diet pill use [21].

But despite the growing evidence linking various forms of the media, including social media, to body image dissatisfaction, very few have examined the role that dating apps play in this relationship [7, 8]. To the best of our knowledge, only one study has examined the association between dating app use and UWCBs [22]. The study, which was limited to a nationwide sample of sexual minority men in Australia and New Zealand, found a positive correlation between dating app use and eating disorder symptoms but no significant association between the two variables [22].

Given dating apps are a form of non-traditional media that provides a digital environment where users are being evaluated based on their physical appearance, we hypothesize dating app users will demonstrate elevated rates of UWCBs compared to non-users. In addition, based on prior research studies documenting disparities in UWCBs across racial and sexual orientation groups [23–26], we suspect that racial and sexual minorities will demonstrate elevated engagement in UWCBs compared to their white and heterosexual/straight counterparts.

## Methods

### Procedures

Researchers at the Harvard T.H. Chan School of Public Health conducted an online survey as part of the Harvard Chan Physical Activity Study. This study was implemented using Amazon Mechanical Turk (MTurk) and has a broader aim of further understanding physical activity in the U.S. population and its relationship with social determinants and social stressors [27]. Study participants enrolled between October 2017 to December 2017 answered questions assessing frequency of dating app use and engagement in UWCBs.

MTurk is a website created and operated by Amazon since 2005 [28]. The website innovatively utilizes the method of crowdsourcing to engage a large number of online users who are registered “MTurk workers” to complete various tasks [29]. There are more than 500,000 registered MTurk workers worldwide, of which the majority are based in the U.S. [28]. Since its conception, various entities – including businesses and researchers – have used MTurk to recruit participants to complete surveys, engage in experiments, and a wide array of other activities [29]. Previous studies have been successful in utilizing MTurk to measure body image estimation and dissatisfaction [30]. For example, Gardner, Brown, and Boice (2012) recruited more than 300 participants through MTurk to complete an online questionnaire that assessed body image satisfaction among men and women. The authors suggest their experience with the crowdsourcing website supported findings from prior research [31] in that MTurk was an innovative source for generating inexpensive data of good quality. Furthermore, prior research suggests that compared to the general population, MTurk participants are younger, of lower socioeconomic backgrounds, and more likely to be LGBTQ-identifying individuals [32–34].

### Participants

Participants eligible for the Harvard Chan Physical Activity study were limited to adult men and women residing in the U.S. who were ages 18–65 years. Additionally, since one of the main goals of the study was to collect participants’ daily number of steps taken while carrying a mobile device, eligibility to complete the survey was limited to those using an iPhone 6 series smartphone or greater (e.g., iPhone 6 s, 7, 8, X). Thus, participants with older versions of the iPhone (before iPhone 6) and other mobile devices were not eligible. Participants received no more than $5 for completing the online survey. Since the number of eligible participants exposed to the online survey is not known, we cannot calculate a response rate. All participants provided informed consent for participating in the study.

To achieve the aims of our study, we focused our analyses on the 1769 participants of the Harvard Chan Physical Activity study who enrolled between October 2017 to December 2017 and answered questions assessing frequency of dating app use and engagement in UWCBs. Among this sample, we excluded 14 people (0.79%) who did not have complete data on any of the variables of interest. Additionally, we excluded 29 people (1.64%) who self-described their sexual orientation identity as “other” as the experiences and health behaviors of these participants may vary from those identifying as gay, lesbian, or bisexual [35]. Our final analytic sample included 1726 participants.

## Measures

### Dating app use

Participants were asked to indicate the frequency, on average, in which they used dating apps (e.g., Tinder, Grindr, Coffee Meets Bagel, etc.) within the past 30 days. Response options were “never, less than once a day, 1-4 times a day, 5 or more times a day.” Due to the small number of participants reporting dating app use as more than once a day, dating app use was made a binary variable indicating non-users and users.

### Unhealthy weight control behaviors

A series of questions assessed participants’ engagement in UWCBs with the purpose of lowering weight or changing their body shape within the past 12 months. These UWCBs included fasting (not eating for at least a day), self-induced vomiting, using laxatives, using diet pills without a doctor’s advice, using anabolic steroids, and using muscle-building supplements (e.g., creatine, amino acids, DHEA, hydroxyl methyl-butyrate [HMB], or growth hormone). Response options were “never, less than once a month, 1-3 times a month, once a week, more than once a week.”

### Demographic information

Participants reported their age in years (18–25, > 25–30, > 30–40, > 40 years), annual household income in U.S. dollars (<$25 K, $25 K- < $50 K, $50 K- < $75 K, $75- < $100 K, >$100 K), sexual orientation identity (heterosexual, gay or lesbian, bisexual, other), sex (male, female), race/ethnicity (White non-Hispanic, White Hispanic, African American, Asian, multiple races/other), marital status (married, never married, divorced, separated, widowed), and height and weight from which body mass index (BMI; kg/m^2^) was calculated. Due to the small number of participants who reported being divorced, widowed, or separated, marital status was categorized as married, never married, or other.

## Statistical analyses

We conducted all statistical analyses in 2018 using Stata 15 and R version 3.4.3. Frequencies and descriptive statistics were examined for all variables. Each of the UWCBs (e.g., fasting, self-induced vomiting, using laxatives, using diet pills, using anabolic steroids, and using muscle-building supplements) were examined independently. Each UWCB was dichotomized such that the response “never” scored 0 and responses “less than once a month,” “1-3 times a month,” “once a week,” and “more than once a week” scored 1. Chi-square tests were used to compare differences in sociodemographic characteristics and the dichotomized UWCBs between dating app users versus non-users among females and males. To achieve our study aims, we conducted a series of multivariate logistic regression models with dating app use as the main predictor, controlling for sex, race/ethnicity, income, age, marital status, sexual orientation, to estimate the odds ratios and 95% confidence intervals of each dichotomized UWCB. We also conducted our analyses separately for women and for men based on prior research findings suggesting gender differences in eating outcomes [36]. Significance level was set at alpha = 0.05 for all tests.

## Results

### Sample characteristics and prevalence of UWCBs

Table 1 presents the prevalence of sociodemographic characteristics and unhealthy weight control behaviors in our sample of app users and non-users (*N* = 1726). Overall, 63.6% (*n* = 1098) of our sample were women and 36.4% (*n* = 628) were men. Among women, the majority were non-dating app users (83.3%, *n* = 915), white (68.6%, *n* = 753), between ages 18 and 30 years (55.8%, *n* = 613), and identified as straight or heterosexual (86.5%, *n* = 950). Men demonstrated similar characteristics as most were also non-dating app users (66.7%, *n* = 419), white (68.0%, *n* = 427), between 18 and 30 years of age (58.8%, *n* = 369), and straight or heterosexual (88.9%, *n* = 558).

**Table 1:** Sample Demographics of Dating App Users and Non-Users (*N* = 1726)

	Females					Males					Total
	n = 1098 (63.6%)					n = 628 (36.4%)					N = 1726 (100.0%)
Characteristic	App Users (*n* = 183)	Non-users (n = 915)	χ^2^	df	*p*-value	App Users (*n* = 209)	Non-users (n = 419)	χ^2^	df	p-value	
Age (years), n (%)			20.99	3	0.0001*			23.55	3		
18–25	74 (40.4)	236 (25.8)				79 (37.8)	101 (24.1)			< 0.0001*	490 (28.4)
> 25–30	52 (28.4)	251 (27.4)				71 (34.0)	118 (28.2)				492 (28.5)
> 30–40	41 (22.4)	276 (30.2)				40 (19.1)	135 (32.2)				492 (28.5)
> 40	16 (8.8)	152 (16.6)				19 (9.1)	65 (15.5)				252 (14.6)
Race, n (%)			4.84	4				11.70	4		
White Non-Hispanic	118 (64.5)	635 (69.4)			0.3045	128 (61.2)	299 (71.4)			0.0197*	1180 (68.4)
Hispanic	18 (9.8)	56 (6.1)				17 (8.1)	29 (6.9)				120 (7.0)
Black	16 (8.7)	93 (10.2)				28 (13.3)	25 (6.0)				162 (9.4)
Asian	17 (9.3)	67 (7.3)				19 (9.1)	34 (8.1)				137 (7.9)
Other or Mixed	14 (7.7)	64 (7.0)				17 (8.1)	32 (7.6)				127 (7.3)
Household Income ($), n (%)			9.15	4				14.26	4		
< 25 k	36 (19.6)	113 (12.3)			0.0574	34 (16.3)	41 (9.8)			0.0065*	224 (13.0)
25 – 50 k	49 (26.8)	268 (29.3)				51 (24.4)	93 (22.2)				461 (26.7)
> 50 – 75 k	47 (25.7)	217 (23.7)				59 (28.2)	98 (23.4)				421 (24.4)
> 75 – 100 k	25 (13.7)	138 (15.1)				33 (15.8)	78 (18.6)				274 (15.9)
> 100 k	26 (14.2)	179 (19.6)				32 (15.3)	109 (26.0)				346 (20.0)
Sexual Orientation, n (%)			7.48	2				11.75	2		
Heterosexual or Straight	147 (80.3)	803 (87.7)			0.0237*	173 (82.8)	385 (91.8)			0.0028*	1508 (87.4)
Bisexual	28 (15.3)	83 (9.1)				17 (8.1)	17 (4.1)				145 (8.4)
Gay or Lesbian	8 (4.4)	29 (3.2)				19 (9.1)	17 (4.1)				73 (4.2)
Marital Status^a^, n (%)			83.58	2				49.17	2		
Never Married	127 (69.4)	358 (39.1)			< 0.0001*	140 (67.0)	176 (42.0)			< 0.0001*	801 (46.4)
Married	30 (16.4)	488 (53.4)				52 (24.9)	227 (54.2)				797 (46.2)
Other	26 (14.2)	69 (7.5)				17 (8.1)	16 (3.8)				128 (7.4)
Fasting for Weight Control, n (%)			30.61	1				43.28	1		
Yes	82 (44.8)	248 (27.1)			< 0.0001*	113 (54.1)	113 (27.0)			< 0.0001*	556 (32.2)
No	101 (55.2)	667 (72.9)				96 (45.9)	306 (73.0)				1170 (67.8)
Vomiting for Weight Control, n (%)			50.58	1				100.15	1		
Yes	41 (22.4)	54 (5.9)			< 0.0001*	76 (36.4)	22 (5.3)			< 0.0001*	193 (11.2)
No	142 (77.6)	861 (94.1)				133 (63.6)	397 (94.7)				1533 (88.8)
Laxative Use, n (%)			30.61	1				100.15	1		
Yes	44 (24.0)	85 (9.3)			< 0.0001*	86 (41.1)	32 (7.6)			< 0.0001*	247 (14.3)
No	139 (76.0)	830 (90.7)				123 (58.9)	387 (92.4)				1479 (85.7)
Diet Pill Use, n (%)			35.22	1				121.53	1		
Yes	49 (26.8)	94 (10.3)			< 0.0001*	84 (40.2)	21 (5.0)			< 0.0001*	248 (14.4)
No	134 (73.2)	821 (89.7)				125 (59.8)	398 (95.0)				1478 (85.6)
Anabolic Steroid Use, n (%)			82.40	1				115.54	1		
Yes	29 (15.8)	13 (1.4)			< 0.0001*	76 (36.4)	16 (3.8)			< 0.0001*	134 (7.8)
No	154 (84.2)	902 (98.6)				133 (63.6)	403 (96.2)				1592 (92.2)
Muscle Building Supplement^b^, n (%)			43.50	1				66.66	1		
Yes	37 (20.2)	50 (5.5)			< 0.0001*	104 (49.8)	76 (18.1)			< 0.0001*	267 (15.5)
No	146 (79.8)	865 (94.5)				105 (50.2)	343 (81.9)				1459 (84.5)

Total dating app users was 392 (46.7% females, 53.3% males). *Indicates a *p*-value < 0.05. Chi-square (χ^2^) tests were used to compare differences between dating app users versus non-users. df = degrees of freedom

^a^Other marital status includes participants who were separated or divorced

^b^Includes products such as creatine, amino acids, DHEA, hydroxyl methylbutyrate [HMB], or growth hormone

UWCBs were prevalent among both women and men, also presented in Table 1. The prevalence of laxative use was 11.7% (*n* = 129) and 18.8% (*n* = 118) among women and men, respectively. Nearly 9% (n = 95) of women and 16% (*n* = 98) of men reported vomiting for weight control. Other prevalent UWCBs include fasting (30.0% of women, *n* = 330; 36.0% of men, *n* = 226), diet pill use (13.0% women, *n* = 143; 16.7% men, *n* = 105), anabolic steroids (4.8% of women, n = 42; 14.6% of men, *n* = 92), and muscle building supplements (7.9% of women, *n* = 87; 28.7% of men, *n* = 180).

Results from chi-square tests (also presented in Table 1) suggest that engagement in each of the six UWCBs of interest in this study was higher among dating app users compared to non-users for both males and females. The distribution of age, marital status, sexual orientation, and BMI were also significantly different between dating app users and non-users in both gender groups. For example, among both females and males, dating app users had a higher proportion of non-married and sexual minority-identifying individuals (e.g., gay or bisexual) compared with non-users; the age distribution was also relatively younger among dating app users compared with non-users among both males and females.

### Relationship between dating apps and UWCBs

Table 2 presents the multivariate logistic regression estimates of the odds of engaging in UWCBs among adults participating in the Harvard Chan Physical Activity study. Dating app users demonstrated significantly elevated odds of all six UWCBs (odds ratios ranged from 2.7 to 16.2) compared to those who were non-users, controlling for sex, race/ethnicity, sexual orientation, income, age, and marital status. Compared to women, the odds of muscle building supplement and steroid use were significantly higher among men. Results also suggest African Americans demonstrated significantly elevated odds of engaging in all six UWCBs compared to white participants. Results did not suggest elevated odds of any UWCB based on sexual orientation identity.

**Table 2:** Odds of UWCBs among adults in the Harvard Chan Physical Activity Study (N = 1726)

OR (95% CI)						
	Vomiting	Laxative Use	Fasting	Diet Pill Use	Muscle Building Supplement Use^a^	Anabolic Steroid Use
Dating App Use						
User	**7.809 (5.415, 11.262)**	**6.284 (4.514, 8.748)**	**2.656 (2.059, 3.425)**	**6.627 (4.766, 9.215)**	**4.474 (3.265, 6.132)**	**16.226 (9.992, 26.347)**
Non-user (ref)	1.0	1.0	1.0	1.0	1.0	1.0
Sex						
Male	1.400 (0.970, 1.906)	1.280 (0.949, 1.728)	1.153 (0.923, 1.439)	0.920 (0.678, 1.250)	**3.975 (2.947, 5.362)**	**3.034 (1.980, 4.650)**
Female (ref)	1.0	1.0	1.0	1.0	1.0	1.0
Race/Ethnicity						
Asian American	**1.772 (1.004, 3.128)**	1.117 (0.645, 1.934)	**1.542 (1.052, 2.260)**	1.386 (0.821, 2.341)	1.376 (0.818, 2.315)	1.575 (0.771, 3.218)
African American	**2.259 (1.362, 3.748)**	**2.699 (1.747, 4.169)**	**1.960 (1.383, 2.777)**	**2.590 (1.673, 4.009)**	**1.920 (1.187, 3.106)**	**3.439 (1.888, 6.264)**
Hispanic	**2.047 (1.149, 3.646)**	1.533 (0.892, 2.637)	1.468 (0.988, 2.181)	1.250 (0.708, 2.207)	**1.760 (1.043, 2.972)**	1.872 (0.900, 3.893)
Other or Mixed	**2.513 (1.471, 4.293)**	1.618 (0.962, 2.723)	1.427 (0.954, 2.135)	**2.610 (1.609, 4.234)**	**2.027 (1.228, 3.345)**	**2.596 (1.338, 5.036)**
White (ref)	1.0	1.0	1.0	1.0	1.0	1.0
Sexual Orientation						
Gay/Bisexual	1.022 (0.639, 1.636)	0.741 (0.470, 1.168)	1.127 (0.824, 1.540)	0.939 (0.611, 1.442)	0.893 (0.577, 1.383)	1.046 (0.590, 1.855)
Heterosexual (ref)	1.0	1.0	1.0	1.0	1.0	1.0
Income (USD)						
> 100 K	0.758 (0.400, 1.435)	0.954 (0.551, 1.653)	**0.501 (0.340, 0.739)**	1.142 (0.659, 1.981)	1.206 (0.699, 2.090)	1.102 (0.507, 2.394)
75-100 K	1.490 (0.827, 2.682)	1.088 (0.629, 1.881)	**0.663 (0.448, 0.980)**	1.369 (0.794, 2.361)	**1.912 (1.122, 3.259)**	**2.095 (1.011, 4.342)**
50- < 75 K	0.963 (0.546, 1.699)	1.072 (0.645, 1.780)	**0.603 (0.421, 0.864)**	1.243 (0.747, 2.070)	1.341 (0.807, 2.228)	0.979 (0.479, 2.000)
25- < 50 K	0.979 (0.564, 1.701)	0.945 (0.573, 1.559)	**0.627 (0.444, 0.884)**	0.998 (0.602, 1.654)	0.866 (0.520, 1.443)	1.010 (0.501, 2.038)
< 25 K (ref)	1.0	1.0	1.0	1.0	1.0	1.0
Age (years)						
> 40	1.777 (1.165, 2.712)	1.347 (0.911, 1.993)	**1.410 (1.059, 1.878)**	1.262 (0.857, 1.860)	1.001 (0.688 1.457)	1.619 (0.960, 2.729)
> 30–40	0.879 (0.533, 1.452)	0.914 (0.587, 1.421)	1.176 (0.858, 1.611)	0.948 (0.613, 1.465)	**0.504 (0.322, 0.788)**	1.012 (0.545, 1.880)
> 25–30	0.636 (0.322, 1.256)	0.985 (0.574, 1.691)	0.906 (0.607, 1.357)	0.819 (0.471, 1.426)	**0.486 (0.275, 0.857)**	1.009 (0.455, 2.236)
18–25 (ref)	1.0	1.0	1.0	1.0	1.0	1.0
Marital Status						
Never married	**0.464 (0.309, 0.697)**	**0.441 (0.306, 0.637)**	**0.729 (0.562, 0.946)**	**0.514 (0.357, 0.741)**	**0.655 (0.457, 0.939**)	**0.557 (0.336, 0.923)**
Other^b^	0.946 (0.500, 1.790)	0.794 (0.452, 1.396)	1.038 (0.678, 1.588)	0.870 (0.494, 1.533)	1.206 (0.699, 2.080)	1.372 (0.647, 2.907)
Married (ref)	1.0	1.0	1.0	1.0	1.0	1.0

Engagement of UWCBs occurred the past 12 months. Boldface indicates statistical significance (*p* < 0.05). CIs = confidence interval

^a^Includes products such as creatine, amino acids, DHEA, hydroxyl methylbutyrate [HMB], or growth hormone

^b^Participants were considered married if they were currently married, and not married if they were never married, divorced, or separated

Tables 3 and 4 present the results of the gender-stratified multivariate logistic regression models for women and men, respectively. Women who use dating apps had 2.3 to 26.9 times the odds of engaging in all six UWCBs compared to women who were non-users. The same trend of elevated odds was found among men. Men who use dating apps had 3.2 to 14.6 times the odds of engaging in all six UWCBs compared to men who were non-users. Results of both gender-stratified analyses also highlighted racial/ethnic disparities as Asian American, African American, Hispanic, and other or mixed participants often demonstrated significantly higher odds of UWCB engagement compared to their white counterparts.

**Table 3:** Odds of UWCBs among women in the Harvard Chan Physical Activity Study (N = 1098)

OR (95% CI)						
	Vomiting	Laxative Use	Fasting	Diet Pill Use	Muscle Building Supplement Use^a^	Anabolic Steroid Use
Dating App Use						
User	**6.442 (3.837, 10.816)**	**4.515 (2.828, 7.210)**	**2.285 (1.611, 3.240)**	**4.011 (2.583, 6.226)**	**5.265 (3.090, 8.973)**	**26.926 (11.512, 62.979)**
Non-user (ref)	1.0	1.0	1.0	1.0	1.0	1.0
Race/Ethnicity						
Asian American	1.028 (0.435, 2.430)	0.809 (0.352, 1.857)	1.412 (0.868, 2.320)	0.743 (0.339, 1.631)	1.341 (0.557, 3.226)	0.713 (0.150, 3.388)
African American	0.935 (0.417, 2.097)	**2.313 (1.322, 4.048)**	**1.808 (1.180, 2.769)**	**1.759 (1.002, 3.090)**	**2.373 (1.163, 4.844)**	**3.665 (1.347, 9.976)**
Hispanic	1.762 (0.848, 3.660)	1.544 (0.780, 2.057)	1.455 (0.871, 2.429)	**2.355 (1.291, 4.295)**	**2.657 (1.275, 5.537)**	**3.413 (1.224, 9.515)**
Other or Mixed	1.120 (0.474, 2.643)	0.999 (0.452, 2.206)	1.164 (0.693, 1.956)	0.719 (0.314, 1.647)	1.643 (0.710, 3.803)	**1.454 (0.375, 5.629)**
White (ref)	1.0	1.0	1.0	1.0	1.0	1.0
Sexual Orientation						
Gay/Bisexual	1.082 (0.587, 1.993)	0.649 (0.352, 1.199)	1.055 (0.719, 1.550)	1.141 (0.684, 1.903)	1.348 (0.735, 2.475)	0.570 (0.199, 1.632)
Heterosexual (ref)	1.0	1.0	1.0	1.0	1.0	1.0
Income (USD)						
> 100 K	1.145 (0.529, 2.475)	0.660 (0.356, 1.223)	0.698 (0.458, 1.064)	0.813 (0.439, 1.505)	**0.367 (0.155, 0.871)**	**0.166 (0.047, 0.587)**
75-100 K	1.150 (0.518, 2.550)	0.754 (0.399, 1.424)	0.659 (0.421, 1.030)	0.919 (0.487, 1.735)	1.255 (0.595, 2.649)	0.417 (0.142, 1.221)
50- < 75 K	1.503 (0.647, 3.492)	0.763 (0.377, 1.540)	0.668 (0.404, 1.103)	1.245 (0.631, 2.457)	1.845 (0.835, 4.078)	0.929 (0.307, 2.811)
25- < 50 K	0.994 (0.413, 2.392)	0.538 (0.261, 1.109)	**0.536 (0.329, 0.875)**	1.038 (0.528, 2.042)	0.717 (0.293, 1.752)	0.573 (0.177, 1.855)
< 25 K (ref)	1.0	1.0	1.0	1.0	1.0	1.0
Age (years)						
> 40	1.236 (0.688, 2.221)	0.901 (0.528, 1.538)	1.306 (0.906, 1.883)	1.059 (0.636, 1.762)	1.392 (0.724, 2.674)	1.442 (0.542, 3.833)
> 30–40	0.584 (0.292, 1.168)	0.669 (0.373, 1.200)	1.135 (0.763, 1.688)	0.838 (0.479, 1.466)	0.884 (0.425, 1.840)	0.961 (0.314, 2.937)
> 25–30	0.690 (0.297, 1.602)	0.744 (0.368, 1.504)	0.776 (0.464, 1.300)	0.887 (0.451, 1.748)	0.800 (0.324, 1.973)	1.489 (0.410, 5.409)
18–25 (ref)	1.0	1.0	1.0	1.0	1.0	1.0
Marital Status						
Never married	**0.348 (0.195, 0.622)**	**0.311 (0.186, 0.522)**	0.746 (0.533, 1.043)	**0.494 (0.305, 0.798)**	**0.471 (0.252, 0.878)**	**0.215 (0.081, 0.569)**
Other^b^	0.519 (0.213, 1.265)	0.626 (0.304, 1.292)	0.907 (0.540, 1.521)	0.728 (0.363, 1.461)	1.234 (0.549, 2.775)	0.685 (0.219, 2.139)
Married (ref)	1.0	1.0	1.0	1.0	1.0	1.0

Engagement of UWCBs occurred the past 12 months. Boldface indicates statistical significance (p < 0.05). CIs = confidence interval

^a^Includes products such as creatine, amino acids, DHEA, hydroxyl methylbutyrate [HMB], or growth hormone

^b^Participants were considered married if they were currently married, and not married if they were never married, divorced, or separated

**Table 4:** Odds of UWCBs among men in the Harvard Chan Physical Activity Study (N = 628)

OR (95% CI)						
	Vomiting	Laxative Use	Fasting	Diet Pill Use	Muscle Building Supplement Use^a^	Anabolic Steroid Use
Dating App Use						
User	**11.738 (6.534, 21.087)**	**9.949 (5.970, 16.580)**	**3.165 (2.159, 4.639)**	**14.437 (8.125, 25.650)**	**4.394 (2.932, 6.585)**	**14.581 (7.861, 27.046)**
Non-user (ref)	1.0	1.0	1.0	1.0	1.0	1.0
Race/Ethnicity						
Asian American	**3.958 (1.691, 9.265)**	1.651 (0.7532, 3.620)	1.851 (1.000, 3.428)	**3.205 (1.423, 7.217)**	1.375 (0.704, 2.687)	2.310 (0.970, 5.500)
African American	**6.795 (3.087, 14.958)**	**3.385 (1.637, 7.000)**	**2.301 (1.233, 4.295)**	**4.683 (2.163, 10.140)**	1.551 (0.803, 2.998)	**4.119 (1.879, 9.031)**
Hispanic	**5.413 (2.310, 12.684)**	2.203 (0.889, 5.457)	1.447 (0.746, 2.810)	**4.025 (1.711, 9.469)**	1.685 (0.838, 3.388)	2.193 (0.882, 5.454)
Other or Mixed	**4.335 (1.806, 10.403)**	**2.581 (1.142, 5.832)**	**2.121 (1.113, 4.042)**	**2.756 (1.133, 6.708)**	1.781 (0.893, 3.554)	2.339 (0.940, 5.822)
White (ref)	1.0	1.0	1.0	1.0	1.0	1.0
Sexual Orientation						
Gay/Bisexual	1.102 (0.509, 2.389)	0.892 (0.435, 1.828)	1.266 (0.730, 2.196)	0.732 (0.330, 1.623)	0.567 (0.303, 1.063)	1.523 (0.726, 3.196)
Heterosexual (ref)	1.0	1.0	1.0	1.0	1.0	1.0
Income (USD)						
> 100 K	0.454 (0.167, 1.235)	2.203 (0.889, 5.457)	**0.431 (0.225, 0.826)**	1.363 (0.510, 3.645)	1.444 (0.734, 2.842)	1.982 (0.678, 5.792)
75-100 K	1.161 (0.471, 2.861)	1.790 (0.712, 4.501)	0.642 (0.334, 1.234)	1.460 (0.554, 3.845)	1.388 (0.693, 2.780)	**3.880 (1.377, 10.932)**
50- < 75 K	0.677 (0.283, 1.619)	1.815 (0.764, 4.313)	**0.502 (0.270, 0.933)**	1.975 (0.804, 4.852)	1.925 (0.928, 3.991)	1.872 (0.685, 5.113)
25- < 50 K	0.755 (0.320, 1.782)	1.663 (0.698, 3.966)	**0.494 (0.268, 0.910)**	1.374 (0.553, 3.416)	1.727 (0.842, 3.543)	**2.810 (1.058, 7.466)**
< 25 K (ref)	1.0	1.0	1.0	1.0	1.0	1.0
Age (years)						
> 40	**3.201 (1.638, 6.254)**	1.396 (0.583, 3.345)	1.216 (0.629, 2.353)	0.647 (0.224, 1.869)	0.825 (0.510, 1.334)	0.669 (0.224, 1.997)
> 30–40	1.841 (0.842, 4.024)	1.469 (0.732, 2.949)	1.289 (0.762, 2.181)	1.399 (0.666, 2.940)	**0.365 (0.204, 0.653)**	1.063 (0.489, 2.313)
> 25–30	**0.483 (0.134, 0.738)**	**2.072 (1.127, 3.809)**	1.587 (0.991, 2.541)	1.664 (0.872, 3.176)	**0.374 (0.178, 0.787)**	1.595 (0.834, 3.049)
18–25 (ref)	1.0	1.0	1.0	1.0	1.0	1.0
Marital Status						
Never married	0.708 (0.378, 1.325)	0.676 (0.387, 1.180)	0.698 (0.456, 1.067)	0.673 (0.366, 1.237)	0.839 (0.531, 1.328)	0.819 (0.436, 1.537)
Other^b^	**3.063 (1.025, 9.155)**	1.323 (0.500, 3.503)	1.603 (0.719, 3.573)	1.628 (0.561, 4.724)	1.357 (0.553, 3.328)	1.703 (0.568, 5.106)
Married (ref)	1.0	1.0	1.0	1.0	1.0	1.0

Engagement of UWCBs occurred the past 12 months. Boldface indicates statistical significance (p < 0.05). CIs = confidence interval

^a^Includes products such as creatine, amino acids, DHEA, hydroxyl methylbutyrate [HMB], or growth hormone

^b^Participants were considered married if they were currently married, and not married if they were never married, divorced, or separated

We also explored the role of BMI as a potential confounder in the relationship between dating app use and UWCBs. Our findings remained statistically significant despite the inclusion of BMI in our multivariate logistic regression models for all six UWCBs (See Additional file 1: Table S1).

## Discussion

Our study adds to the limited public health literature on UWCBs and their association with the use of dating apps – an increasingly popular form of nontraditional media that is believed to be a contributor of body dissatisfaction [22]. To our knowledge, this is the first study to investigate the association between dating app use and UWCBs among U.S. adults. Specifically, we hypothesized dating app users would demonstrate elevated engagement of UWCBs, such as self-induced vomiting, fasting, and diet pill use. Such behaviors are not medically recommended for weight loss and are considered clinically relevant symptoms of eating disorders [37]. Our results supported this hypothesis. First, our analyses revealed a high prevalence of various UWCBs among the men and women in our study - ranging from self-induced vomiting for weight control to anabolic steroid use. Additionally, our results documented a higher prevalence of the six UWCBs among dating app users than non-users in our study. These findings may be a result of the image- and appearance-centered culture of dating apps as users attempt to find sexual and/or romantic partners; such claims, however, warrant additional study. Consistent with existing literature, we documented elevated rates of UWCBs among racial/ethnic minorities [38, 39]. For example, a prior study of nearly 17,000 U.S. adolescents found that the odds of UWCBs were elevated 2–10 times in most ethnic groups relative to whites [39]. This finding partially supported our second hypothesis that dating app users belonging to racial/ethnic and sexual minority groups would demonstrate higher rates of UWCBs. Our results highlighted racial/ethnic disparities as African Americans reported higher odds of UWCBs compared to their white counterparts. We also documented elevated engagement in many UWCBs among Asian American, Hispanic and other or mixed dating app users. We did not, however, find elevated odds of UWCBs based on sexual orientation. Prior research have found sexual minority men to be at greater risk for eating disorders, such as anorexia and bulimia nervosa, compared with heterosexual men [40–42]; studies also suggest that sexual minority men place high priority on physical attractiveness and thinness [43, 44], as well as increased desire for muscularity [45]. .

With the tremendous growth in their usage in the U.S. [1], and an increasing number of studies linking their use to body image concerns and UWCBs, there is an urgency to further understand how dating apps influence health behaviors and outcomes.. And while these apps allow users to communicate with each other, and often privately, prior studies suggest this avenue of digital communication has proliferated interpersonal discrimination, such as racism and weight shaming [11].

According to the Tripartite Influence model [46, 47], appearance pressures from peers, parents, and the media lead to body image dissatisfaction and UWCBs [46]. Dating apps, arguably another form of modern-day social media, often contain commercial ads and user profiles depicting images conveying societally accepted image ideals for men and women. Thus, as with other forms of media, users of dating apps may internalize such societal appearance ideals and possibly compare their own appearance to those that they see – two processes that the Tripartite Model posits lead to body image dissatisfaction and ultimately eating disturbances [48, 49]. Therefore, future studies, particularly those executing a longitudinal design, ought to apply the framework of the Tripartite Model by exploring the role of peers, family, and other media in the relationship between dating app use and UWCBs.

Overall, our study has several limitations for consideration. The cross-sectional design of the study and absence of long-term assessment of dating app use limited our ability to establish temporal or causal relationships between dating apps and UWCBs. It is possible that individuals already engaging in UWCBs may be drawn to using dating apps, and that dating app use in turn could exacerbate disordered eating behavior symptoms. Our cross-sectional study cannot disentangle these different plausible pathways but highlights the need for additional studies (e.g., cohort or quasi-experimental) to identify the causal links between dating app use and UWCBs.. In addition, the results of the online survey used in this study relied on self-reported data and did not collect indicators of psychosocial factors, such as experiences with weight stigma, body image concerns, self-esteem, and depression, which may be possible mediating variables in the relationship between body dissatisfaction and UWCBs [50]. Our findings are also limited in regards to generalizability as participants were restricted to U.S.-based participants in possession of an iPhone 6 series or greater, who may have differing sociodemographic characteristics (e.g., income) from those with other mobile devices [51]. In addition, MTurk workers are not necessarily representative of the general population (e.g., overrepresentation of women) [52].. The online survey did not assess the types and brands of dating app services used by our participants, as some may have less tolerance for appearance-based discrimination among users [53]. For instance, multiple dating app services began imposing profile changes and interventions intended to minimize discrimination as well as promote inclusivity on their platforms in fall 2018. The dating app “Scruff,” which is geared towards men who have sex with men, no longer requires ethnicity to be listed on user profiles and began sending in-app messages to users who display “racial language” on their profiles [54]. Lastly, we did not assess participants’ motivation for dating app use (e.g., to find romantic partners, sexual partners, and/or friends). Such information could further explain the possible relationship between dating app use and UWCBs.

## Conclusions

This study contributes to the limited literature by exploring the association between dating app use and UWCBs. Whether the use of dating apps can be attributed to adverse health outcomes, including UWCBs, remains unclear. The findings from our study, however, continue to fuel speculations that dating app users may be at risk of preventable physical and mental health outcomes. Therefore, identifying individuals at risk of eating disorders and their risk factors is critical in informing effective public health efforts aimed at alleviating the global burden of these potentially deadly yet preventable conditions. Based on our findings, we recommend future studies aim to assess the association between dating app use and UWCBs temporally and use a more representative sample. Such studies should specifically explore the underlying mechanisms as to how and why dating app use may contribute to UWCBs and possibly the development of eating disorders.

## Additional file

Additional file 1: **Table S1.** Adjusted Odds of UWCBs among adults in the Harvard Chan Physical Activity Study (*N* = 1726)* (PDF 25 kb)

## Abbreviation

UWCBs: Unhealthy weight control behaviors
