# Supplementary material for: Dating app use and unhealthy weight control behaviors among a sample of U.S. adults: a cross-sectional study
Source: J Eat Disord. 2019 May 31;7:16. doi: 10.1186/s40337-019-0244-4 (PMC6543621; doi:10.1186/s40337-019-0244-4)
Supplement: Supplementary file 1 — Table S1. Adjusted Odds of UWCBs among adults in the Harvard Chan Physical Activity Study (N = 1726)* (PDF 25 kb) [file 40337_2019_244_MOESM1_ESM.pdf]

Table S1. Adjusted Odds of UWCBs among adults in the Harvard Chan Physical Activity Study (N=1,726)*

	OR (95% CI)					
	**Vomiting**	**Laxative Use**	**Fasting**	**Diet Pill Use**	**Muscle Building Supplement Use[a]**	**Anabolic Steroid Us[illegible]**
**Dating App Use**						
User	**7.652 (5.302, 11.044)**	**6.331 (4.551, 8.808)**	**2.546 (1.992, 3.255)**	**6.824 (4.904, 9.495)**	**4.227 (3.128, 5.713)**	**13.003 (8.237, 20.52[illegible]**
Non-user (ref)	1.0	1.0	1.0	1.0	1.0	1.0

Note: *Models adjusted for sex, race/ethnicity, sexual orientation, income, age, marital status, and body mass index. Boldface indicates statistical significance ($p<0.05$). CIs = confidence interval.

[a]Includes products such as creatine, amino acids, DHEA, hydroxyl methylbutyrate [HMB], or growth hormone.
